# Mediastinal pulmonary artery is associated with greater artery diameter and lingular division volume

**DOI:** 10.1038/s41598-017-01384-1

**Published:** 2017-04-28

**Authors:** Hitoshi Dejima, Yusuke Takahashi, Tai Hato, Katsutoshi Seto, Tetsuya Mizuno, Hiroaki Kuroda, Noriaki Sakakura, Masafumi Kawamura, Yukinori Sakao

**Affiliations:** 1 Department of Thoracic Surgery, Aichi Cancer Center Hospital, 1-1 Kanokoden, Chikusa, Nagoya, Aichi Japan; 2 Department of General Thoracic Surgery, Teikyo University School of Medicine, 2-11-1 Kaga, Itabashi, Tokyo Japan; 3 Department of General Thoracic Surgery, Keio University School of Medicine, 31 Shinanomachi, Shinjuku, Tokyo Japan

## Abstract

Pulmonary vessels have numerous variation and aberrant branching patterns. Mediastinal lingular artery (MLA), the most common aberrant branch, might contribute to greater blood flow to lingular division. Hence, we investigated a correlation between lingular division volume and MLA using three-dimensional CT volumetry. We included 199 consecutive patients who underwent surveillance chest CT to detect possible malignancies in April 2015. We measured lingular division volume and cross-sectional area of lingular arteries using three-dimensional CT volumetry. MLA was identified in 58 cases (29.1%). The MLA group had significantly greater lingular division volume (median ± quartile deviation: 378.3 ± 75.5 mL vs. 330.0 ± 87.5 mL; p = 0.021) and percentage lingular division to left lung volume (19.0 ± 2.62% vs. 16.6 ± 2.39%; p < 0.001) than the non-MLA group. Total cross-sectional area of lingular arteries of the MLA group was significantly larger than that of the non-MLA group (46.1 ± 9.46 vs. 40.2 ± 5.76 mm^2^; p = 0.003). The total cross-sectional area of the lingular arteries strongly correlated to the percentage of lingular division to left lung volume (r = 0.689, p < 0.001). This is the first report demonstrating a positive correlation between branching pattern of pulmonary artery and lung volume.

## Introduction

Complex lung architecture is often compared to a tree, which has intricate cellular structure generated by extremely elaborate process. Pulmonary vessels thus have numerous variation and aberrant branching patterns^1–3^. Particularly, the prevalence of the variant and aberrant branching is higher in left lung and it is well known that the most common variation is a mediastinal lingular artery (MLA)^2–4^. In recent years, three-dimensional computed tomography (3D-CT) combined with computer-aided diagnosis (CAD) has become widely used that can provide us accurate identification of peripheral branches of pulmonary vessels including intersegmental veins for simulation of surgery including segmentectomy^5^. In addition, this allows us to easily identify intersegmental plane as well as to calculate lung segment volume with automated algorithm. MLA may have greater diameter than interlobar lingular artery (ILA) as MLA is usually the first branch of the left main pulmonary artery. Thus, we hypothesized that MLA can largely affect lingular division volume via greater blood flow. In this study, we aimed to investigate relationship between lingular division volume and MLA using 3D-CT with CAD.

## Results

**Figure 1:**
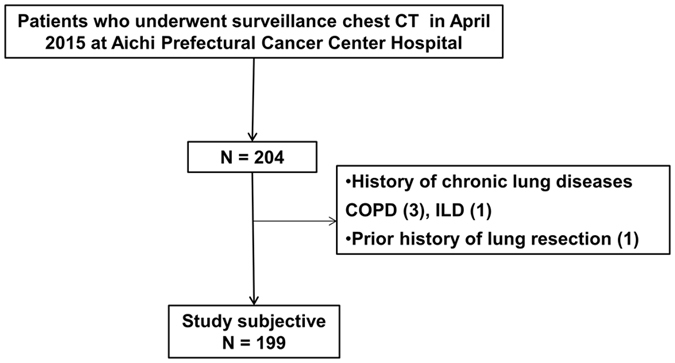
A patient selection chart shows that 204 patients who underwent surveillance chest CT to rule out possible intrathoracic malignancies in April 2015 at Aichi Cancer Center Hospital were included. Among them, 3 patients with history of COPD, 1 patient with history of ILD, and 1 patient with history of prior lung resection were excluded. The remaining 199 patients were selected as our study cohort.

**Figure 2:**
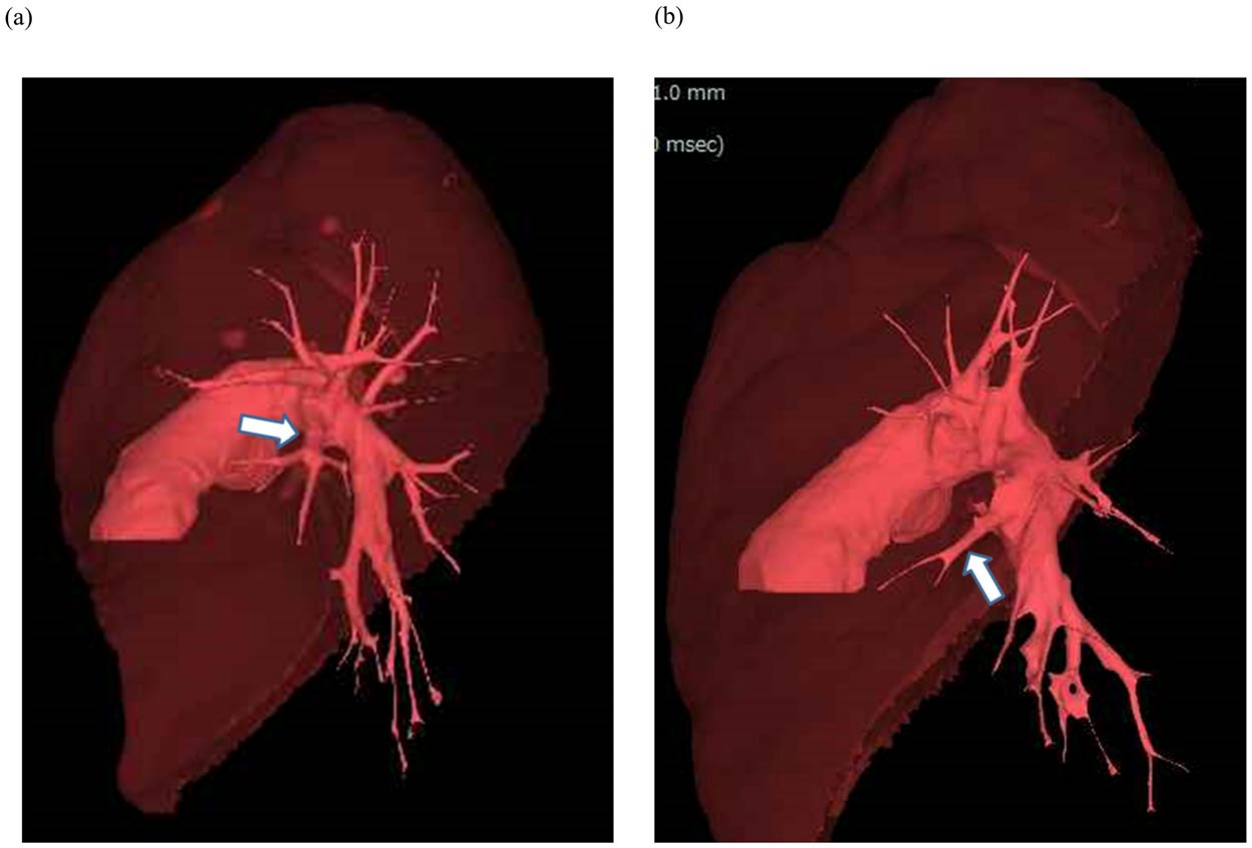
(**a**) A case of the “mediastinal lingular artery (MLA) group”. Three-dimensional computed tomography shows the MLA which branched as the first branch of the left main pulmonary artery (arrow). (**b**) A case of the “non-MLA group”mediastinal lingular artery”. Three-dimensional computed tomography shows theinterlobar lingular artery (ILA) which branched from interlobar portion of the left pulmonary artery (arrow).

Among 204 patients who underwent surveillance chest CT, three patients with of medically treated chronic obstructive pulmonary disease (COPD), one patient with interstitial lung disease (ILD) and one patient with history of pulmonary resection were excluded. Finally, a total 199 patients with medical record and HRCT data were analyzed (Fig. 1). No nodular lesions obstructing bronchi of greater than subsegment were detected on the CT. No patients were excluded due to infiltrative attenuation or pleural effusion. As shown in Table 1, our cohort included 113 men (56.8%) and 86 women (43.2%) with a median age of 64.0 years (range: 25–84 years). Ninety patients (45.2%) were former/current smokers. MLA was identified in 58 patients (29.1%; Fig. 2a) who were classified as “MLA group”. Among them 20 patients had one MLA and 38 patients had one MLA and one ILA, whereas there were no cases with two MLAs. In “non-MLA group”, 90 had one ILA, 51 had two ILAs (Fig. 2b). Number of lingular arteries identified was 1 in 110 cases (55.3%) and 2 in 89 cases (44.7%). A median diameter of lingular artery was 7.5 mm (range: 2.3–14.5 mm).

**Table 1:** Characteristics of the study cohort (n = 199).

**Age**	
Median (range)	64.0 (25.0–84.0)
**Gender**	
male	113
female	86
**Smoking history**	
Never-smoker	109
Smoker	90
**Body Mass Index**	
Median (range)	21.2 (16.4–35.7)
**Smoking Index (pack-years**)	
Median (range)	10.0 (0.00–150)
**History of Cardiovascular diseases**	
Non	190
Effort angina	5
Paroxysmal supraventricular tachycardia	2
Atrioventricular block	1
Aortic valve stenosis	1
**Number of lingular segment artery**	
1	110
2	89
**Branching pattern of lingular artery**	
Mediastinal type × 1	20
Mediastinal type × 2	0
Mediastinal type × 1 + interlobar type × 1	38
Interlobar type × 1	90
Interlobar type × 2	51
**Diameter of lingular artery (mm**)	
Median (range)	7.5 (2.3–14.5)

As shown in Table 2, there were no significant differences in clinical data including age, sex, smoking index, CT-CTR, height, body weight, body mass index, Goddard classification, and percent low-attenuation area (%LAA) in each lung between the two groups. Lingular division volume in the MLA group was significantly larger than that in the non-MLA group (378.3 ± 75.5 mL vs. 330.0 ± 87.5 mL; p = 0.021). The MLA group showed significantly greater lingular division/total lung volume compared with the non-MLA group (8.81 ± 1.26% vs. 7.72 ± 1.03%; p < 0.001). When compared with the non- MLA group, the MLA group showed significantly greater lingular division/left lung volume (19.0 ± 2.62% vs. 16.6 ± 2.39%; p < 0.001) as well as lingular division/left upper lobe volume (36.0 ± 4.00% vs. 31.0 ± 4.00%; p < 0.001).

**Table 2:** Correlation between presence of mediastinal lingular artery and clinicopathological factors in the original cohort.

Factors	Non-MLA group (n = 141)	MLA group (n = 58)	*p-v*alue*
**Age (years**)	65.0 ± 6.5	64.0 ± 10.5	0.802
**Sex**			
Female	64 (45%)	22 (38%)	0.334
Male	77 (55%)	36 (62%)	
**Smoking index (pack-years**)	8.0 ± 15.0	7.6 ± 16.8	0.582
**Cardiothoracic ratio (%**)	44.3 ± 3.00	43.6 ± 3.50	0.401
**Height (cm**)	160.9 ± 7.00	161.5 ± 6.50	0.602
**Weight (kg**)	54.0 ± 6.00	56.7 ± 7.50	0.252
**Body mass index (kg/m** ^2^)	20.7 ± 2.50	21.6 ± 2.00	0.592
**Goddard classification**			
<1	140 (99%)	57 (98%)	0.514
1–2.5	1 (1%)	1 (2%)	
≧2.5	0 (0%)	0 (0%)	
**Total lung LAA (%**)	0.10 ± 0.00	0.10 ± 0.50	0.879
**Left lung LAA (%**)	0.10 ± 0.00	0.10 ± 0.50	0.760
**LUL lung LAA (%**)	0.10 ± 0.50	0.10 ± 0.50	0.698
**Total lung volume (mL**)	4324 ± 676.7	4208 ± 678.8	0.374
**Right lung volume (mL**)	2309 ± 378.5	2318 ± 353.0	0.639
**Left lung volume (mL**)	2036 ± 332.5	1959 ± 326.0	0.259
**LUL volume (mL**)	1091 ± 180.5	1069 ± 172.0	0.313
**Lingular division volume (mL**)	330.0 ± 87.5	378.3 ± 75.5	0.021
**Lingular division/total lung (%**)	7.72 ± 1.03	8.81 ± 1.26	<0.001
**Lingular division/Left lung (%**)	16.6 ± 2.39	19.0 ± 2.62	<0.001
**Lingular division/LUL (%**)	31.0 ± 4.00	36.0 ± 4.00	<0.001

Continuous variables were represented as median with a quartile deviation. *Fisher’s exact test for categorical variables and Mann-Whitney U test for continuous variables, MLA = mediastinal lingular artery, LAA = low attenuation area, LUL = left upper lobe.

Next, we investigated the correlation between the lingular division/left lung volume and continuous variables such as age, smoking index, CT-CTR, body mass index. The lingular division/left lung volume was not correlated with either age (r = 0.019, p = 0.729; Fig. S1a), body mass index (r = 0.107, p = 0.188; Fig. S1b), smoking index (r = 0.126, p = 0.118; Fig. S1c), or CT-CTR (r = 0.012, p = 0.884; Fig. S1d).

**Figure 3:**
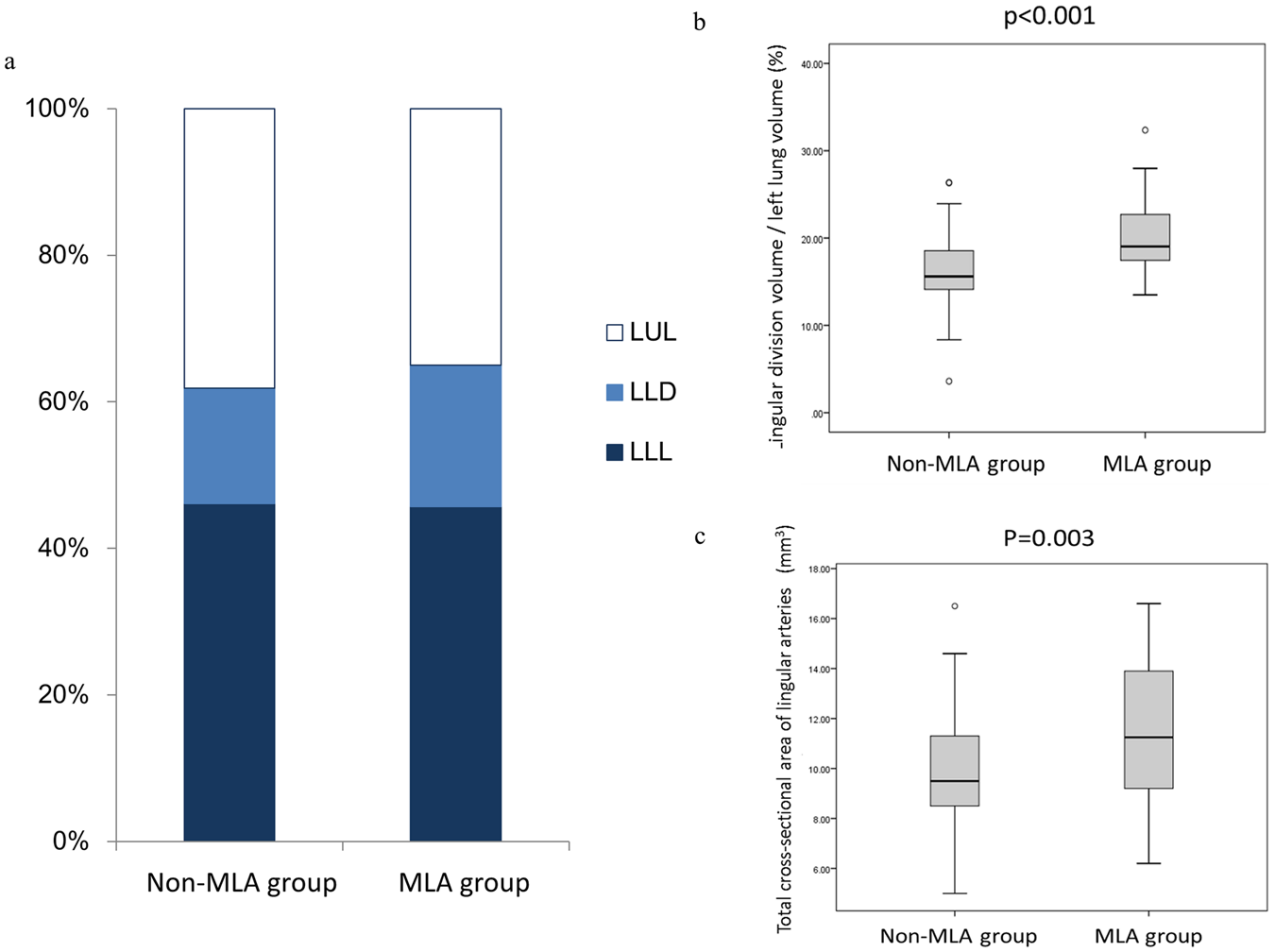
(**a**) The proportion of left upper division (LUD), left lingular division (LLD), and left lower lobe (LLL) to let lung volume in the original cohort. The MLA group showed significantly greater lingular division/left lung volume as well as left upper division/left lung volume compared with the non-MLA group (p < 0.001 and p = 0.003, respectively). Left lower lobe/left lung volume was not statistically different between the two groups (p = 0.925). (**b**) After matching, lingular division/left lung volume in the MLA group is significantly greater than that of the non-MLA group (p < 0.001). (**c**) Total cross-sectional area of lingular arteries in the MLA group is significantly greater than that in the non-MLA group (p = 0.003) in the matched cohort.

We then conducted case-control matching analysis to further minimize influence of possible confounders. As shown in Table 3, there were no significant differences in patient characteristics except for lingular division volume. The lingular division volume in the MLA group was larger than in the non-MLA group (378.3 ± 75.5 mL vs. 323.6 ± 84.5 mL; p = 0.028). The lingular division/total lung volume in the MLA group was also significantly greater than that in the non-MLA group and (8.81 ± 1.26% vs. 7.72 ± 1.03%; p < 0.001). Figure 3a showed percentage of either left upper division, left lingular division, or left lower lobe to left lung volume. There were no statistical differences in left lower lobe/left lung volume between the MLA and non-MLA groups (45.0 ± 4.67% vs. 45.9 ± 3.43%; p = 0.925). The MLA group showed significantly higher lingular division/left lung volume compared to the non-MLA group (19.0 ± 2.62% vs. 16.6 ± 2.22%; p < 0.001; Fig. 3b), while left upper division/left lung volume in the MLA group is significantly smaller than that in the non-MLA group (34.5 ± 4.26% vs. 38.0 ± 3.93%; p = 0.003). When compared with the non-MLA group, the MLA group had significantly higher lingular division/left upper lobe lung volume (36.0 ± 4.00% vs. 30.0 ± 4.50%; p < 0.001).

**Table 3:** Correlation between presence of mediastinal lingular artery and clinicopathological factors in matched analysis.

Factors	Non-MLA group (n = 58)	MLA group (n = 58)	*p-v*alue *
**Age (years**)	63.0 ± 6.0	64.0 ± 10.5	0.875
**Sex**			
Female	22 (38%)	22 (38%)	1.000
Male	36 (6%)	36 (62%)	
**Smoking index (pack-years**)	7.8 ± 14.3	7.6 ± 16.8	0.803
**Cardiothoracic ratio (%**)	43.7 ± 3.02	43.6 ± 3.50	0.726
**Height (cm**)	160.9 ± 6.50	161.5 ± 6.50	0.609
**Weight (kg**)	55.5 ± 5.50	56.7 ± 7.50	0.288
**Body mass index (kg/m** ^**2**^)	21.5 ± 2.00	21.6 ± 2.00	0.713
**Goddard classification**			
<1	57 (98%)	57 (98%)	1.000
1–2.5	1 (2%)	1 (2%)	
≧2.5	0 (0%)	0 (0%)	
**Total lung LAA (%**)	0.10 ± 0.00	0.10 ± 0.50	0.628
**Left lung LAA (%**)	0.10 ± 0.00	0.10 ± 0.50	0.934
**LUL lung LAA (%**)	0.10 ± 0.50	0.10 ± 0.50	0.473
**Total lung volume (mL**)	4558 ± 593.7	4208 ± 678.8	0.242
**Right lung volume (mL**)	2293 ± 314.5	2318 ± 353.0	0.487
**Left lung volume (mL**)	2101 ± 344.0	1959 ± 326.0	0.331
**LUL volume (mL**)	1091 ± 167.0	1069 ± 172.0	0.204
**Lingular division volume (mL**)	323.6 ± 84.5	378.3 ± 75.5	0.028
**Lingular division/total lung (%**)	7.34 ± 1.10	8.81 ± 1.26	<0.001
**Lingular division/Left lung (%**)	15.6 ± 2.22	19.0 ± 2.62	<0.001
**Lingular division/LUL (%**)	30.0 ± 4.50	36.0 ± 4.00	<0.001

Continuous variables were represented as median with a quartile deviation. *Fisher’s exact test for categorical variables and Mann-Whitney U test for continuous variables, MLA = mediastinal lingular artery, LAA = low attenuation area, LUL = left upper lobe.

**Figure 4:**
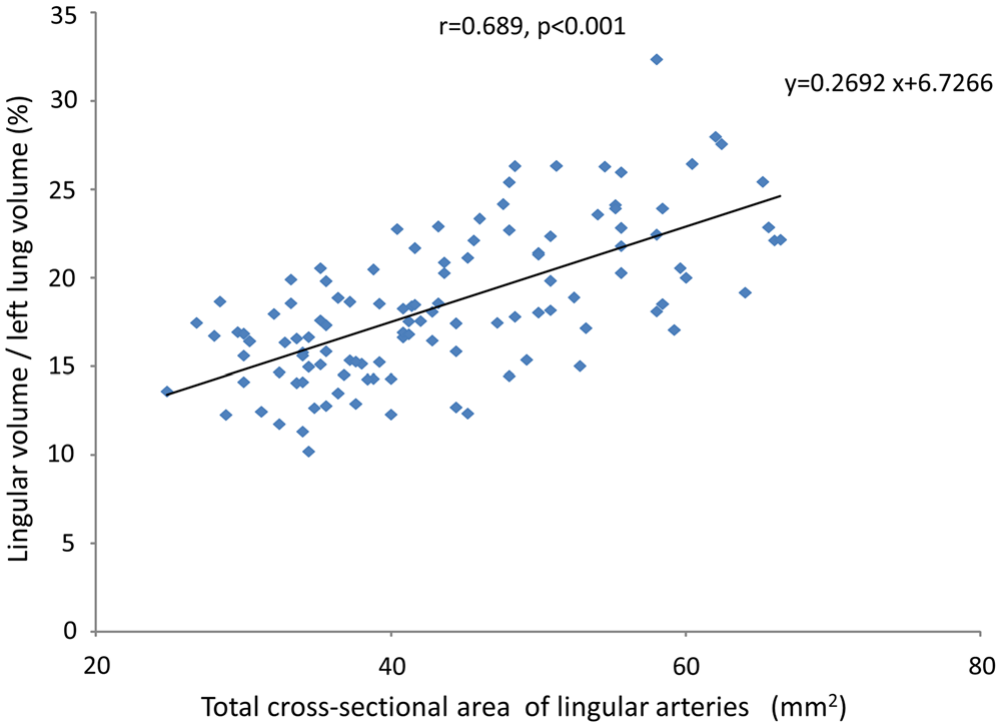
A strong positive correlation between lingular division/left lung volume and total cross-sectional area of lingular arteries is shown by Spearman’s rank correlation test (r = 0.689, p < 0.001).

We further investigated total cross-sectional area of lingular arteries on 3D-CT in the matched cohort. The total cross-sectional area of lingular arteries in the MLA group was significantly greater than that in the non-MLA group (46.1 ± 9.46 vs. 40.2 ± 5.76 mm^2^; p = 0.003; Fig. 3c). In addition, we assessed correlation between lingular arteries and lingular division/left lung volume. As shown in Fig. 4, there was a strong positive correlation between the total cross-sectional area of lingular arteries and the lingular division volume/left lung volume (r = 0.689, p < 0.001).

## Discussion

We demonstrated that the lingular division volume was highly correlated with the branching patterns of lingular arteries as well as the cross-sectional area of lingular arteries using the novel 3D-volumetry analyzing system. In addition, lingular division/left lung volume showed strong correlation to the total cross-sectional area of lingular arteries. Lingular division/left lung volume is considered to be a better index than lingular division/total lung volume because of less variability, while they both showed significant differences between the presence and absence of MLA. To our knowledge, this is the first report investigating a correlation between the branching patterns of pulmonary arteries and lung segment volume.

In this study, the 3D-CT analysis was used to measure the lingular division volume and cross-sectional area of lingular arteries. This semi-automated measurement provided us highly reproducible anatomical data with high interobserver consistency as shown in Fig. S2. Since postoperative liver function is highly predicable by remnant liver volume, liver 3D-volumetry (so-called “virtual hepatectomy”) has been documented^6–9^. On the other hand, it has been used mainly to identify segmental bronchi and vessels such as intersegmental veins for preoperative evaluation of lung segmentectomy^5, 10^, as variations and aberrant branches of pulmonary vessels greatly affect technical difficulty and operative risk. In addition, “number of segment” method that calculates postoperative pulmonary function based on number of resected and remaining segments has been a gold standard to predict postoperative pulmonary function with or without adjustment by perfusion scintigraphy^11, 12^. Although one study reported usefulness of 3D-volumetry to evaluate lung volume for lung transplantation^13^, there is no previous literature investigating correlation between lung segment volume and segment artery branching patterns using 3D-CT volumetry to date. Thus, we should focus on the population with relatively healthy lung to validate our method. On the other hand, it is clinically significant to analyze patients with COPD and/or ILD because previous literature demonstrated structural changes of pulmonary arteries in these patients^14–18^. As shown in Fig. S3, our correlation analysis showed no significant correlation between lingular division/left lung volume and either Goddard classification score or %LAA in both the non-MLA and MLA groups. This suggests that influence of unrecognized COPD represented by Goddard score or %LAA can be ignored in this cohort. If decreased pulmonary artery flow is associated with decreased normal lung volume, it might be relevant to progression mechanisms of COPD and/or ILD. Lingular division may tend to be smaller in COPD patients who had been excluded from our study cohort, whereas influence of lingular artery type seems to be smaller than the current study cohort. Regardless of branching patterns of lingular arteries, the lingular artery dimension was likely to be associated with the lingular artery volume in the excluded 4 patients. Further study will clarify correlation between pulmonary artery flow and lung volume in patients with COPD and/or ILD. In addition, the current findings in normal population might also suggest a different role of pulmonary artery flow in normal lung development as below. The most important finding of our study is that the lingular division volume is significantly associated with the branching patterns and cross-sectional area of lingular arteries. MLA, the most common variation, was reportedly found in approximately 25% of healthy population^1–3^, which is consistent with that we identified 29.6% cases have one or two MLAs in our relatively healthy population. In previous reports, lung volume was affected by various factors including age^19^, sex^20^, smoking status^19^, CTR^21^, and body mass index^22, 23^, all of which were matched to further minimize possible effects on lung volume. Pulmonary emphysema is also characterized by the lung hyperinflation due to peripheral small airway obstruction^24, 25^. Thus, we excluded patients with history of COPD and additionally evaluate emphysema on HRCT as %LAA to focus on the analysis of normal lung. On the other hand, there is a small possibility that not only CTR but also biased heart position can affect left lung volume. Therefore, measuring lingular division/left lung volume can be a better way to evaluate lingular division volume.

It is quite reasonable that centrally branched arteries have greater cross-sectional area which may contribute to higher blood flow to corresponding lung parenchyma. Regarding correlation between blood flow and organ volume, portal blood flow was associated with degree of liver regeneration after hepatectomy^26^ as well as after liver transplantation^27^. Our finding is more likely to be collateral evidence that blood flow can be a determinant of lung volume in normal lung development. However, there might be the following major concerns for this speculation: (1) whether cross-sectional area of lingular arteries does adequately reflect the blood flow and (2) whether the correlation between the cross-sectional area of lingular arteries and lingular volume might not be a cause-effect relationship. Physiologically, blood flow is directly proportional to the twice power of cross-sectional area of blood vessels if blood pressure is constant based on the Hagen-Poiseuille equation^28^. Individual differences of pulmonary artery pressure among healthy population are quite small^29^. Our study population did not include patients with chronic lung diseases which can cause secondary pulmonary hypertension. Because hypoxic pulmonary vasoconstriction is specifically seen when unilateral lung ventilation in general anesthesia^30^, it is unnecessary to consider it in the current setting. At this point, it is reasonable to consider that blood flow and total cross-sectional area of the lingular arteries can reflect blood flow in our population. On the other hand, we cannot directly measure blood flow of peripheral pulmonary arteries in a non-invasive way. Thus, the relationship between lingular division volume and blood flow should be speculative. Also, we do not have appropriate analytical methods and any data to answer the second question above. There are no studies investigating correlation between lingular volume and diameter of lingular arteries based on long-term changes of lung volume during lung development from birth to adolescent. Originally, relatively high prevalence of variation and aberrant branches of pulmonary vessels and bronchi are attributed to the complexity of the lung development. Normal lung development starts with formation of lung bud from ventral foregut during gestational fourth week^31^. After development of tracheal primordia and two main bronchi, the lung bud grows into adjacent splanchnic mesoderm where branching of vessels and bronchi are repeatedly induced during gestational period. Pulmonary vasculature is developed in parallel to bronchial tree. Even postnatally, increase of number of alveoli by septation of alveolar saccules continues to adolescence. During this period, vasculogenesis and remodeling of the capillary is essential for maturation of functioning alveoli. Lung parenchyma which consists largely of alveoli and alveolar duct is mainly generated in this “septation” process with regulation by angiogenic factors including vascular endothelial growth factor (VEGF)^32^. VEGF signaling are also essential for alveolarization which morphologically resembles “septation” in regeneration process after pneumonectomy in mice^33–35^. In addition, VEGF expression is significantly associated with better long-term compensatory restoration of pulmonary function after major lung resection^36^. Thus, some researchers consider that blood flow of pulmonary arteries can affect lung volume in some ways. Of note, we cannot clarify which of lingular volume or total cross-sectional area of lingular arteries is cause of the other, even if there is causal relationship. Nonetheless, our findings may shed a ray of light on the controversy whether pulmonary artery is an active determinant for normal lung development, which is of a great interest for researchers in this uncertain field.

The current findings using 3D volumetry are also useful for surgeon for better understanding of detailed lung anatomy. Lung segmentectomy has often been performed in patients with peripheral small-sized non-small cell lung cancer, even though the survival and functional benefit is still controversial. Relationship between vasculature and organ development will become more important in context toward regenerative medicine.

Limitations of our study include selection biases due to the retrospective nature. Although we identified one or more lingular arteries in all patients, possible influence of undetectable small arteries due to the limited spacial resolution of 3D-CT^37^ cannot be denied. In addition, our findings should be validated using the measurement by pulmonary perfusion scan and ventilation scan, which could provide regional quantitative data of blood flow and ventilation^38–40^. Thus, further study is required to confirm the correlation with these reliable modalities.

In conclusion, this is the first report demonstrating a significant influence of branching patterns and cross sectional area of pulmonary arteries on lung volume using novel 3D-CT volumetry, which shed a light on a role of vasculature in lung development.

## Methods

### Subjects and imaging data

This study was approved by Aichi Cancer Center Hospital Ethical Committee. Our study was performed in full accordance with the local IRB guidelines (No. 2015–1–247). Informed consent was obtained from all patients. Our study included 204 consecutive patients who underwent surveillance chest CT to rule out possible intrathoracic malignancies in April 2015 at Aichi Cancer Center Hospital. Patients who met the following criteria were excluded: 1) history of chronic pulmonary diseases, 2) recognizable pleural effusion or massive infiltrative attenuation on chest CT, 3) history of pulmonary resection and/or chest irradiation, 4) medically treated cardiac dysfunction, or 5) significant findings of emphysema, bronchiectasis, and/or bronchial wall thickening on high-resolution CT as previously described^41^.

**Figure 5:**
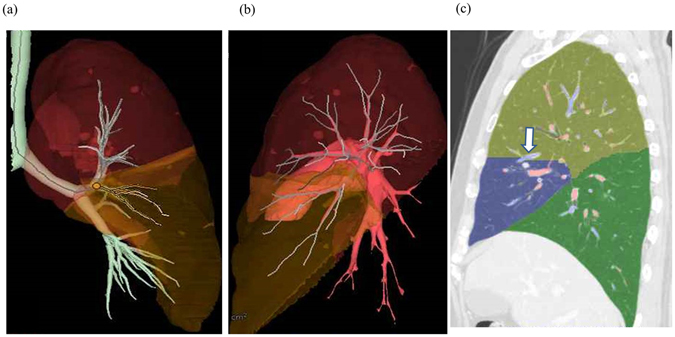
(**a** and **b**) According to the 3D-CT images, lingular bronchi (B4 + 5) and segmental arteries (A4 + A5) are identified. Subsequently, the perfusion area is depicted using an algorithm based on the direction and diameter of the bronchi and artery. (**c**) Visualization of intersegmental plane defined by intersegmental veins (V3a and V3b).

CT scan was performed using 64-detector-row CT scanner (Light Speed VCT; General Electric, CT, USA). Whole chest was scanned during a breath-hold at deep inspiration phase in supine position, and 1.25-mm thick high-resolution images were reconstructed using standard spatial-frequency reconstruction algorithm. Digital imaging and communications in medicine data was transferred to the commercially available workstation (Synapse Vincent; Fujifilm Medical Co., Tokyo, Japan). 3D images including pulmonary vessels, tracheo-bronchial trees, and lung parenchyma were reconstructed. Lingular division arteries (A4 and A5) and intersegmental veins (V3a and V3b), as well as lingular bronchi (B4 and B5) were identified using the images. Lingular division was isolated on the 3D-images based on the following process^5^: (1) calculation of the bronchial ventilation area and lingular artery perfusion area using an algorithm based on the direction and diameter of the bronchi and artery (Fig. 5a and b), and (2) visualization of intersegmental planes defined by intersegmental veins (V3a and V3b; Fig. 5c). Volumetry was automatically performed using the 3D images^6^. Measurement was performed twice by two experienced operator (HD and KS) and the best value was applied for analysis. Definition of MLA is a branch to lingular division as the first branch of the left main pulmonary artery (Fig. 2a). Normal lingular artery, branches from interlobar left pulmonary artery is called “ILA (interlobar artery)” (Fig. 2b). If cases had at least one MLA, they were categorized to MLA group. A diameter and cross-sectional area of each lingular artery were measured at the bifurcation (total 298 arteries). In case with two lingular arteries, cross-sectional area of the two arteries was totaled per subject.

For evaluation of emphysematous lung, %LAA, so-called “percent emphysema”, was defined as percentage of total voxels within the lung field that fell below −950 Hounsfield units^42–44^ based on 3D-CT analysis. Additionally, Goddard classification was also used to evaluate severity of pulmonary emphysema^45^. CT-based cardiothoracic ratio (CT-CTR) was measured as previously described^46^ because CT-CTR was highly concordant to CTR on chest X-ray.

### Statistical analysis

Categorical variables were represented as counts with percentages. Continuous variables which did not show normal distribution were represented as a median with a quartile deviation (QD) or a range. Independent categorical data were compared by Pearson χ^2^ test and continuous data were compared by Mann-Whitney *U*-test. Correlation was tested using Spearman’s rank correlation. To further minimize the effect of measurable selection bias, a matching procedure was conducted. Variables which reportedly correlate to lung volume, including age^19^, sex^20^, smoking status^19^, CTR^21^, and body mass index^22, 23^ were matched 1 to 1, randomly choosing cases from the set of all possible matches. All tests were two-sided and *p* < 0.05 was considered to be significant. Statistical analysis was performed using SPSS (ver. 23; SPSS Inc., Chicago, IL).

## Electronic supplementary material

Fig. S1a, Fig. S1b, Fig. S1c, Fig. S1d, Fig. S2, Fig. S3a, Fig. S3b, Fig. S3c, Fig. S3d
