# Supplementary material for: Mediastinal pulmonary artery is associated with greater artery diameter and lingular division volume
Source: Sci Rep. 2017 Apr 28;7:1273. doi: 10.1038/s41598-017-01384-1 (PMC5430727; doi:10.1038/s41598-017-01384-1)
Supplement: Supplementary file 1 — Fig. S1a, Fig. S1b, Fig. S1c, Fig. S1d, Fig. S2, Fig. S3a, Fig. S3b, Fig. S3c, Fig. S3d [file 41598_2017_1384_MOESM1_ESM.pdf]

Mediastinal pulmonary artery is associated with greater artery diameter and lingular division volume

Hitoshi Dejima[1,2,+], Yusuke Takahashi[2,*], Tai Hato[3,+], Katsutoshi Seto[1,+], Tetsuya Mizuno[1,+], Hiroaki Kuroda[1,+], Noriaki Sakakura[1,+], Masafumi Kawamura[2,+], Yukinori Sakao[1,+]

[1] Department of Thoracic Surgery, Aichi Cancer Center Hospital, 1-1 Kanokoden, Chikusa, Nagoya, Aichi, Japan

[2] Department of General Thoracic Surgery, Teikyo University School of Medicine, 2-11-1 Kaga, Itabashi, Tokyo, Japan

[3] Department of General Thoracic Surgery, Keio University School of Medicine, 31 Shinanomachi, Shinjuku, Tokyo, Japan

Fig. S1

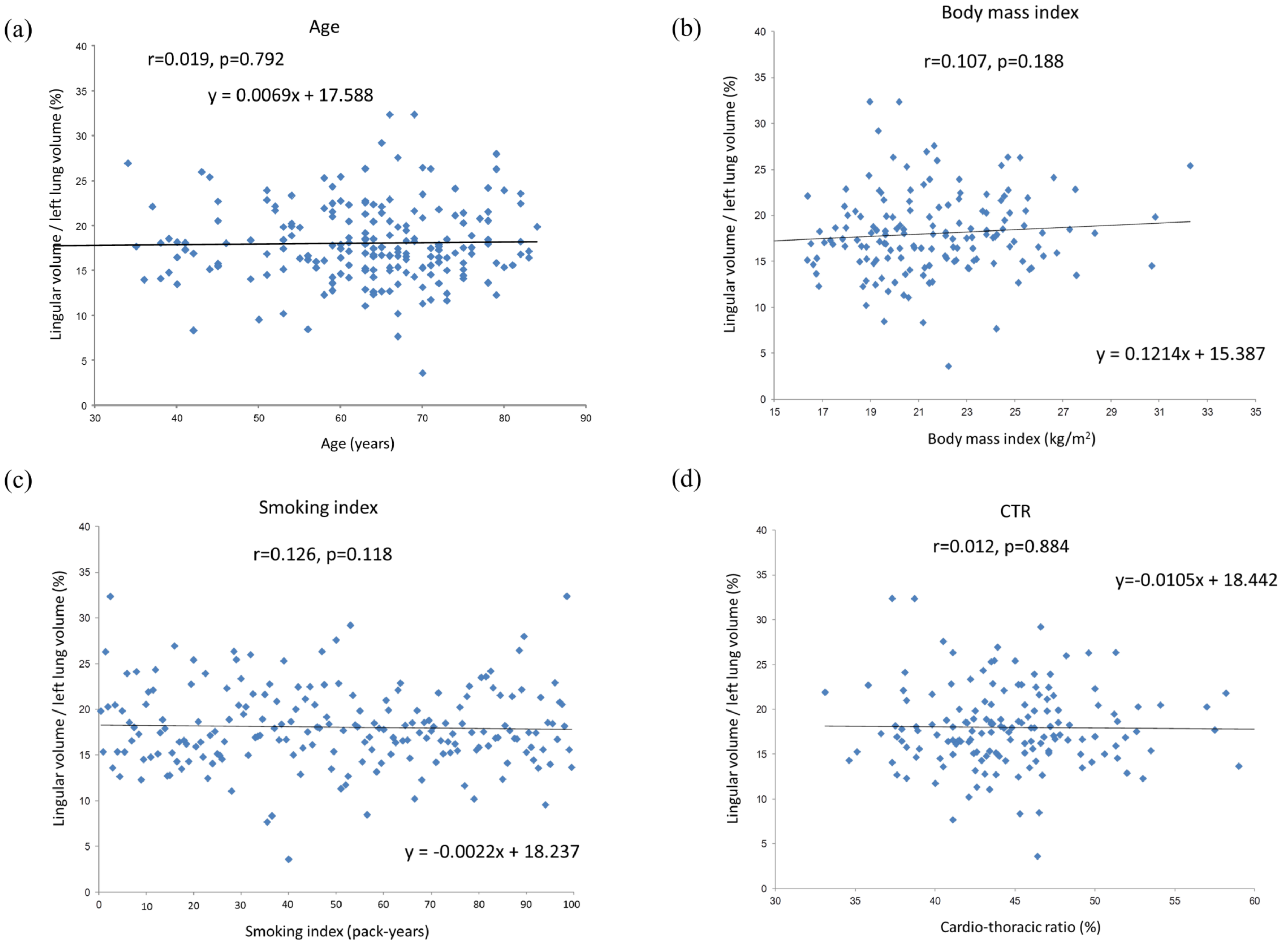
(a)
Age
r=0.019, p=0.792
y = 0.0069x + 17.588
Lingular volume / left lung volume (%)
Age (years)
(b)
Body mass index
r=0.107, p=0.188
y = 0.1214x + 15.387
Lingular volume / left lung volume (%)
Body mass index (kg/m²)
(c)
Smoking index
r=0.126, p=0.118
y = -0.0022x + 18.237
Lingular volume / left lung volume (%)
Smoking index (pack-years)
(d)
CTR
r=0.012, p=0.884
y=-0.0105x + 18.442
Lingular volume / left lung volume (%)
Cardio-thoracic ratio (%)

Fig. S2

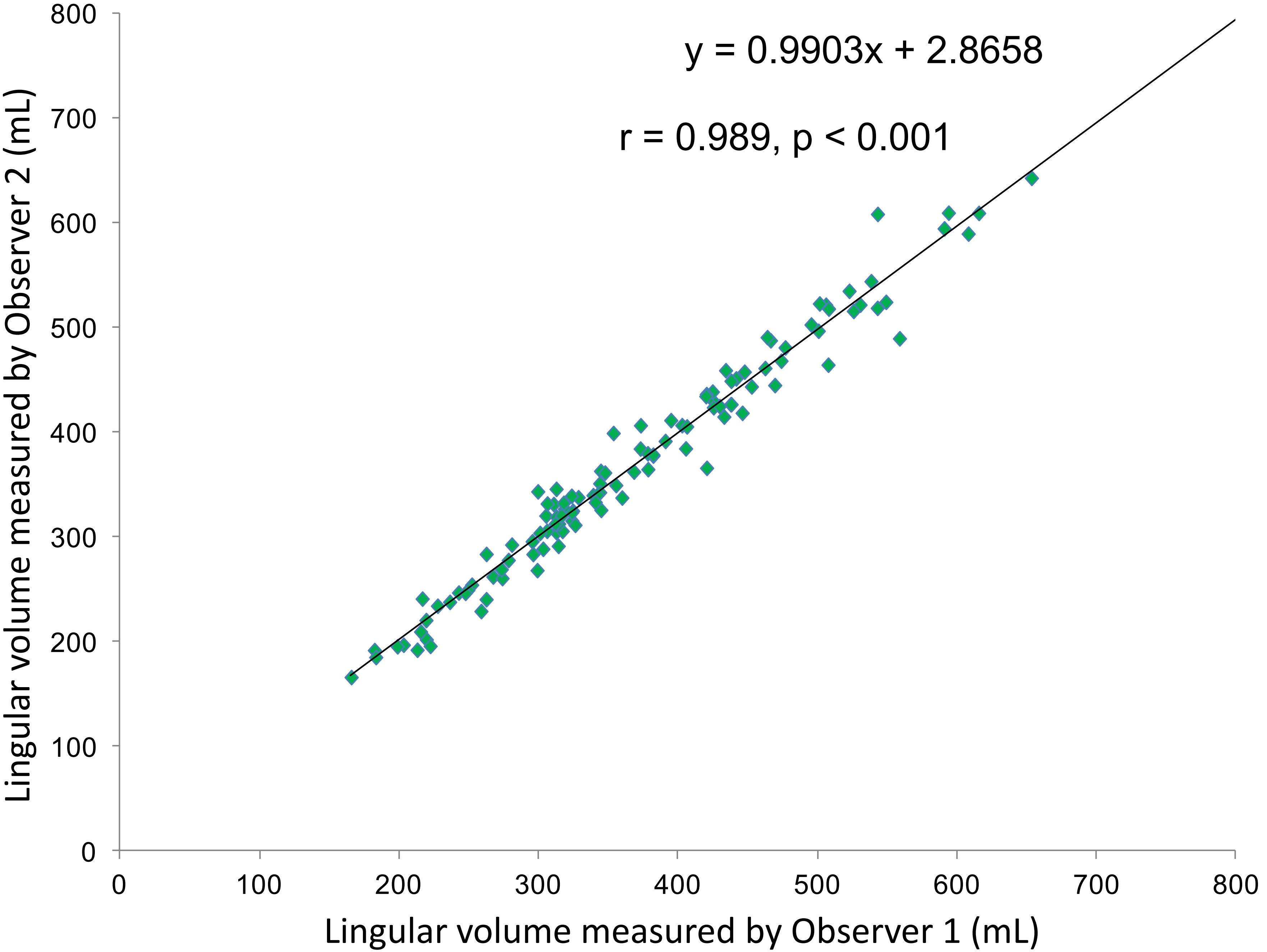

y = 0.9903x + 2.8658
r = 0.989, p < 0.001
Lingular volume measured by Observer 2 (mL)
Lingular volume measured by Observer 1 (mL)
0
100
200
300
400
500
600
700
800

Fig. S3

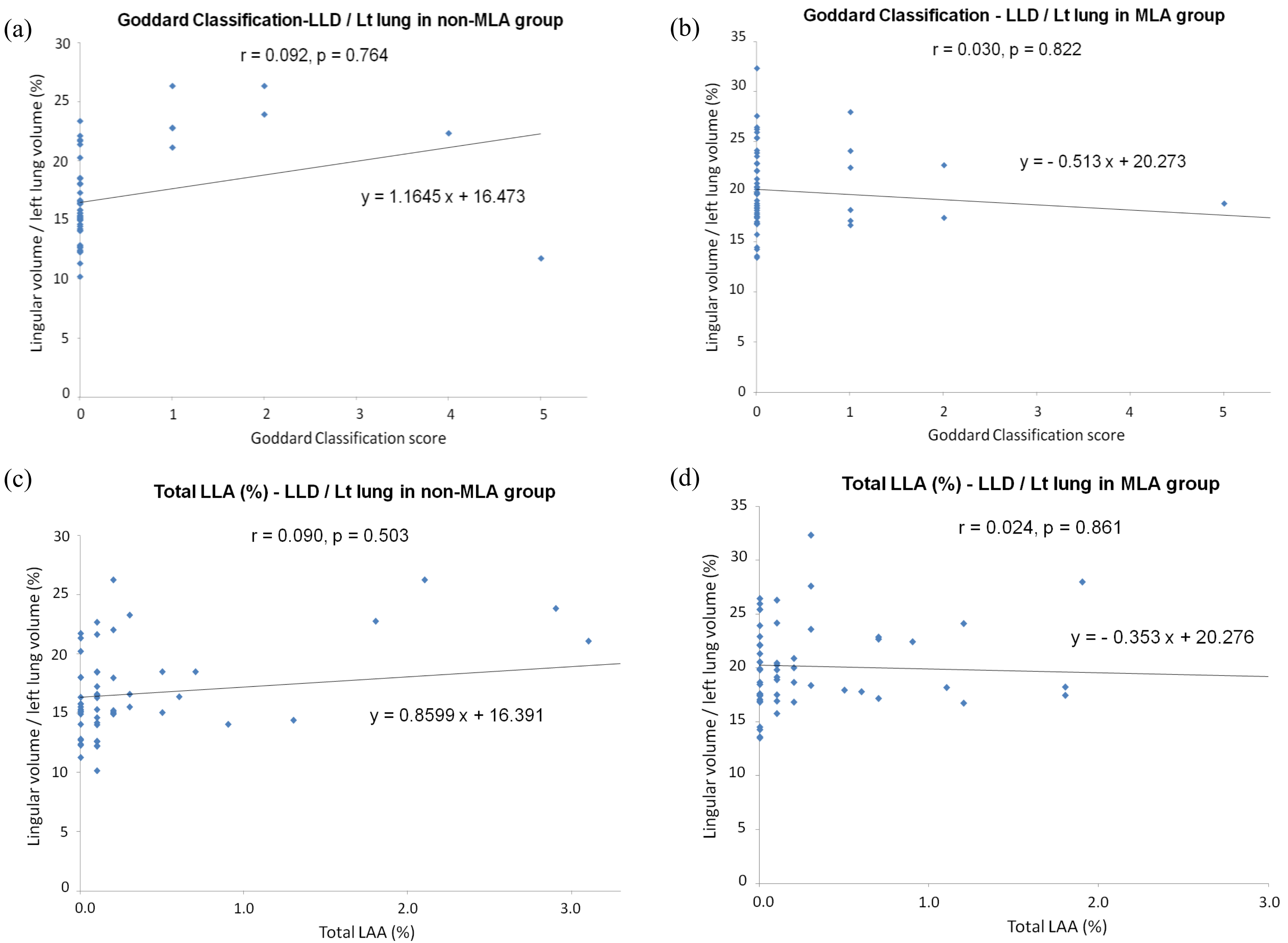
(a)
Goddard Classification-LLD / Lt lung in non-MLA group
r = 0.092, p = 0.764
y = 1.1645 x + 16.473
Lingular volume / left lung volume (%)
Goddard Classification score
(b)
Goddard Classification - LLD / Lt lung in MLA group
r = 0.030, p = 0.822
y = - 0.513 x + 20.273
Lingular volume / left lung volume (%)
Goddard Classification score
(c)
Total LLA (%) - LLD / Lt lung in non-MLA group
r = 0.090, p = 0.503
y = 0.8599 x + 16.391
Lingular volume / left lung volume (%)
Total LAA (%)
(d)
Total LLA (%) - LLD / Lt lung in MLA group
r = 0.024, p = 0.861
y = - 0.353 x + 20.276
Lingular volume / left lung volume (%)
Total LAA (%)

Figure Legends

- Fig. S1

The lingular division/left lung volume shows no significant correlation with either age (r = 0.019, p = 0.729; Fig. S1a), body mass index (r = 0.107, p = 0.188; Fig. S1b), smoking index (r = 0.126, p = 0.118; Fig. S1c), or CT-CTR (r = 0.012, p = 0.884; Fig. S1d).

- Fig. S2

Spearman’s rank correlation test shows a strong positive correlation between lingular division volume measured by Observer 1 and that measured by Observer 2 (r= 0.989, p<0.001).

- Fig. S3

The lingular division/left lung volume is not correlated with Goddard classification score in both the non-MLA group(r = 0.092, p = 0.764; Fig. S1a) and MIA group (r = 0.030, p = 0.822; Fig. S1b). Total %LAA does not correlate to the lingular division/left lung volume in both the non-MLA group (r = 0.090, p = 0.503; Fig. S1c) and MLA group (r = 0.024, p = 0.861; Fig. S1d).
